# Enhancing treatment of lumbar disc herniation with Erxian decoction and auricular acupoint pressure: A randomized controlled trial

**DOI:** 10.1097/MD.0000000000038899

**Published:** 2024-07-12

**Authors:** Wei Feng, Xixi Du, Yuxin Zhao

**Affiliations:** a Department of Rehabilitation, Shangqiu Hospital of Traditional Chinese Medicine, Shangqiu, China; b Department of Orthopedics, Shangqiu Hospital of Traditional Chinese Medicine, Shangqiu, China.

**Keywords:** auricular acupoint pressure, Erxian decoction, lumbar disc herniation, Traditional Chinese Medicine

## Abstract

**Objective:**

The incidence of lumbar disc herniation (LDH) is on the rise annually, with an emerging trend of affecting younger age groups. This study aims to investigate the clinical effectiveness of combining Erxian decoction with auricular acupoint pressure therapy in treating LDH. Our objective is to furnish evidence supporting the incorporation of traditional Chinese medicine (TCM) rehabilitation techniques in clinical settings.

**Methods:**

This randomized controlled trial enrolled 102 patients diagnosed with LDH and allocated them into Control and Intervention groups. The Control group underwent a 2-week rehabilitation regimen, whereas the Intervention group received an augmented treatment comprising Erxian decoction along with auricular acupoint pressure therapy based on the Control group. Main outcome measures included 3 scales – visual analog scale (VAS), Japanese Orthopedic Association (JOA), and Oswestry Disability Index – as well as 3 inflammatory markers: C-reactive protein (CRP), interleukin-6 (IL-6), and tumor necrosis factor-α (TNF-α). Additionally, pressure pain threshold and pain tolerance threshold values were evaluated. Participants were assessed at baseline, on 14-day, and on 28-day posttreatment.

**Results:**

After 2 weeks of treatment, both the Control and Intervention groups exhibited significant improvements in the VAS, JOA, ODI, CRP, IL-6, TNF-α, pressure pain threshold, and pain tolerance threshold (*P* < .05). These improvements persisted at the 28-day in the VAS, JOA, and ODI scores (*P* < .05). On 14-day, the Intervention group showed significantly better outcomes compared to the Control group in terms of the VAS, JOA, ODI, CRP, TNF-α, and pressure pain threshold (*P* < .05).

**Conclusion:**

Compared to conventional rehabilitation therapy, the combination of Erxian decoction and auricular acupoint pressure therapy demonstrates clear benefits in alleviating symptoms in patients with LDH. This approach offers fresh perspectives and substantiates evidence for future treatment strategies in managing LDH.

## 1. Introduction

Lumbar disc herniation (LDH) is characterized by degeneration of the lumbar intervertebral disc, often involving partial or complete rupture of the annulus fibrosus. Such protrusions may irritate or compress adjacent nerves, causing low back and leg pain among other primary symptoms.^[1,2]^ In adult males, the prevalence of LDH exceeds 8%, notably higher than the 2.5% prevalence in females.^[2]^ LDH significantly impairs individuals’ capacity to work, making it a major global health issue.^[3,4]^ Risk factors for LDH include family history, lumbar load, and hard labor.^[5]^ Treatment options to alleviate symptoms of LDH encompass both surgical and nonsurgical methods.^[6]^ Surgical treatment effectively alleviates nerve compression by the nucleus pulposus, but the structural damage from surgical resection may lead to persistent or recurrent low back pain in some patients.^[6–9]^ While analgesic drugs, glucocorticoids, and neurotrophic medications can partially prevent or alleviate residual symptoms, their overall efficacy is limited.^[10,11]^

Traditional Chinese Medicine (TCM) plays a vital role in conservative treatments for pain relief and has amassed considerable experience in managing LDH.^[12–14]^ Erxian Decoction, a traditional Chinese herbal formula, consists of 6 herbs: Epimedii Folium (Yinyanghuo), Curculiginis Rhizoma (Xianmao), Morindae Officinalis Radix (Bajitian), Anemarrhenae Rhizoma (Zhimu), Phellodendri Chinensis Cortex (Huangbai), and Angelicae Sinensis Radix (Danggui).^[15,16]^ Erxian Decoction is frequently utilized for women experiencing endocrine disorders, as well as conditions affecting the immune, nervous, and skeletal systems.^[17,18]^ It has demonstrated effectiveness in both the prevention and treatment of osteoporosis, significantly alleviating associated pain symptoms.^[17,19]^

Auricular pressure therapy, a type of micro-acupuncture, influences the entire body by targeting specific acupoints or pressure areas in the ear.^[20]^ The analgesic mechanisms of this therapy involve modulating pain receptors, inhibiting nerve impulses at the pain site, promoting endorphin secretion, and increasing the pain threshold.^[21,22]^ Numerous studies have conclusively demonstrated the effectiveness of auricular point pressure in alleviating various types of pain, such as postfracture pain,^[23]^ low back pain,^[24]^ and menstrual cramps.^[25]^ Due to its prolonged treatment duration, minimal pain, and low risk of infection compared to traditional acupuncture, auricular acupressure has gained widespread popularity as a complementary therapy, resulting in high patient acceptance.

## 2. Materials and methods

### 2.1. Study design

The study was a randomized, parallel-group, controlled experiment that enrolled patients with LDH in Shangqiu Hospital of Traditional Chinese Medicine between May 2023 and October 2023. The experimental protocols of our study were in strict compliance with the Declaration of Helsinki and received approval from the Ethics Committee of Shangqiu Hospital of Traditional Chinese Medicine (No. SR-2023-4-23-6).

### 2.2. Randomization and masking

**Figure 1.**
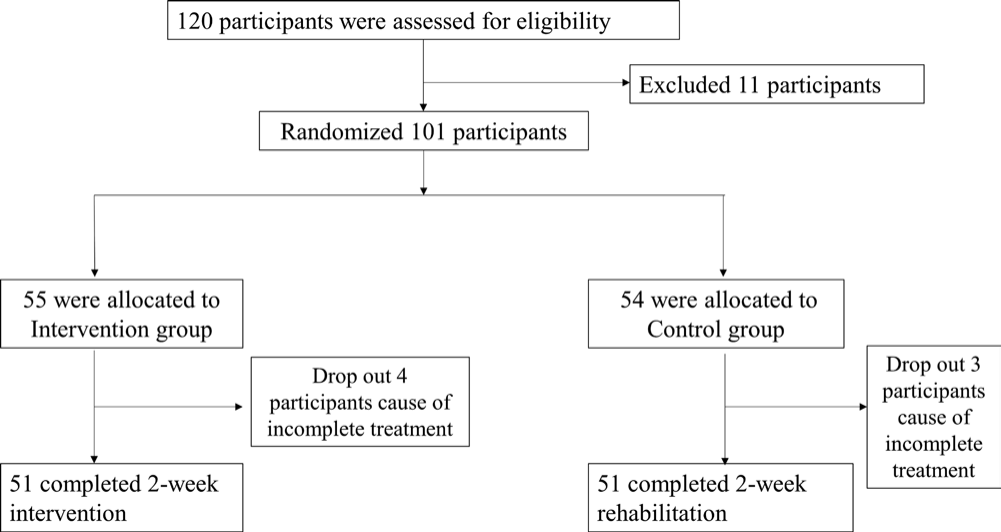
One hundred ten participants were initially recruited. However, 11 participants were excluded due to health issues, leaving 109 participants. These were then randomly assigned to either the Control group (n = 55) or the Intervention group (n = 54). During the study, 4 participants from the Intervention group and 3 from the Control group were excluded for not completing the full treatment course, leading to their withdrawal. Consequently, 51 participants in each of the Intervention and Control groups completed the study.

Randomization was achieved using numbers generated by software, which were then placed in opaque envelopes. These envelopes were opened only after subjects agreed to participate. Participants were randomly at a 1:1 ratio allocated into 2 groups: Intervention group and Control group. The individual assessing outcomes was not involved in either the randomization process or the intervention procedures. Figure 1 illustrates that 102 eligible participants were randomly allocated to either the Intervention group or the Control group.

Control group received standard rehabilitation as outlined in the Chinese Expert Consensus on Rehabilitation of LDH.^[26]^ Intervention group was administered both auricular acupoint pressure with beans and Erxian decoction, alongside the conventional treatment followed by Control group.

### 2.3. Inclusion and exclusion criteria

Inclusion criteria: symptoms of low back and leg pain with imaging-confirmed LDH diagnosis; meeting qi stagnation and blood stasis syndrome; age between 16 and 65 years; and voluntary participation in the clinical trial. Exclusion criteria: low back pain due to other diseases; history of long-term use of analgesic or sedative-hypnotic drugs; co-existing spinal bone lesions, such as spinal fractures or congenital spinal malformations; and pregnant women or those planning pregnancy.

### 2.4. Intervention

In Control group, they performed standardized rehabilitation exercises for 2 weeks, adhering to the Chinese Expert Consensus on Rehabilitation of LDH.^[26]^ The treatment included: lumbar spine traction: patients were positioned horizontally with the chest and pelvis secured. The traction force was adjusted based on the patient’s weight. The optimal traction strength was determined when the patient experienced pain relief and a sense of comfort. Each session lasted for 20 minutes, one time a day; interferential electrostimulation of intermediate frequency: This involved using the HB-ZP2 device for interferential electrostimulation with intermediate-frequency pulses. Electrode pads were cross-placed on both sides of the lumbar region, and the current intensity was adjusted to a level tolerable by the patient. Each session also lasted for 20 minutes, one time a day. The treatment lasted for 2 weeks.

**Figure 2.**
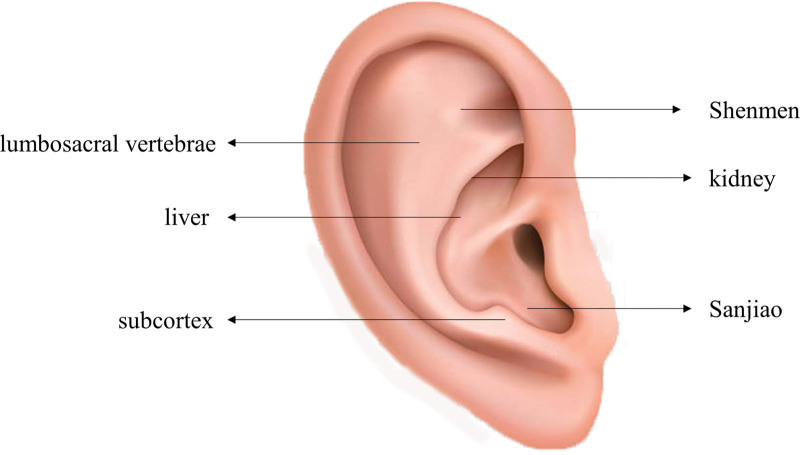
Location of auricular acupoint for pressure therapy.

Intervention group received a combined treatment of auricular acupoint pressure therapy and Erxian decoction, in addition to the Group A protocol. The Erxian decoction formulation included Epimedii Folium (15 g), Curculiginis Rhizoma (15 g), Morindae Officinalis Radix (12 g), Anemarrhenae Rhizoma (6 g), Phellodendri Chinensis Cortex (6 g), and Angelicae Sinensis Radix (12 g). Patients took one dose daily, split into morning and evening, consuming 100 mL each time. The regimen included a 1-day break after 6 consecutive days, defining a week as 1 treatment course. This treatment was administered for 2 courses in total. The auricular acupoint pressure bean therapy targeted Shenmen, Sanjiao, lumbosacral vertebrae, subcortex, liver, and kidney acupoints (Fig. 2), using Semen vaccariae (Wang-Bu-Liu-Xing) seeds as pressure beans. Each acupoint received pressure for approximately 5 minutes, 4 times daily: morning, midday, evening, and bedtime. The therapy was alternated between ears and continued for 2 weeks.

### 2.5. Outcome measures

Scale assessments: the visual analog scale (VAS)^[27]^ assessed patient low back pain intensity, ranging from 0 to 10 points, with lower scores indicating less pain; lumbar spine function was evaluated using the Japanese Orthopedic Association (JOA)^[28]^ assessment scale, which includes subjective symptoms (9 points), physical signs (6 points), and daily activity capability (14 points), cumulatively totaling 29 points. Lower scores reflect poorer lumbar function; the Oswestry Disability Index (ODI)^[29]^ was utilized to assess low back pain and lumbar functionality. The ODI comprises 10 sections, each with 6 options scored between 0 and 5 points, where higher scores denote more severe dysfunction. The assessments using the scales were conducted at 3 time points: pretreatment (baseline), posttreatment (14 days after the initial assessment), and postdischarge (28 days after the initial assessment).

Inflammation indicators: (1) Serum inflammatory markers, including C-reactive protein (CRP), interleukin-6 (IL-6), and tumor necrosis factor-alpha (TNF-α) levels, were measured. Fasting blood samples were collected from patients in the early morning before and after treatment. Post-centrifugation at 3500 rpm, the supernatant was preserved and analyzed using ELISA. They were conducted at pretreatment (baseline) and posttreatment (14 days after the initial measurement).

Pressure pain threshold and pain tolerance threshold: pain thresholds in the lumbar region were assessed using a pressure algometer (Jtech Medical Commander Echo pressure algometer, Jtech Medical, Midvale, UT)^[30]^ while patients were in a prone position. Pressure was applied at a rate of 1 kg/s. The pressure pain threshold was determined as the point where the patient first experienced pain, and the pain tolerance threshold was defined as the point where the pain became intolerable. The 2 values were conducted at pretreatment (baseline) and posttreatment (14 days after the initial measurement).

### 2.6. Statistical analysis

Data analysis was performed using SPSS 23.0 software. Quantitative data, including Inflammation indicators, pressure pain threshold, and pain tolerance threshold, along with scale data adhering to a normal distribution, were presented as mean (±SD). Non-normally distributed data were expressed using quartiles. Pretreatment and posttreatment comparisons of these metrics were conducted using repeated measures ANOVA, with a *P* value (after Bonferroni correction) < .05 indicating statistical significance.

## 3. Results

### 3.1. Basic information

Our study encompassed 102 participants, with 51 each in the intervention and control groups. The demographic and clinical characteristics, including age, gender, pain duration, BMI, employment status, and education level, were no significant differences between the 2 groups (*P* > .05) (Table 1).

**Table 1:** Demographic and clinical characteristics.

Characteristic	Intervention group	Control group	*t*/χ^2^	*P* value
Age (yr)	43.06 ± 13.33	41.22 ± 13.62	0.69	.491
Gender (n)				
Male	26	29	0.35	.552
Female	25	22		
Pain duration (yr)	2.81 ± 1.98	2.89 ± 2.20	0.21	.835
BMI (kg/m^2^)	23.23 ± 3.02	22.92 ± 2.71	0.54	.591
Employment status (n)				
Employed	37	33	5.36	.069
Unemployed	9	17		
Retirement	5	1		
Educational level (n)				
Secondary school and below	18	19	1.35	.510
Universities and colleges	24	27		
Postgraduate	9	5		

### 3.2. Scale assessments of the 2 groups

There were no significant differences in the VAS, JOA, and ODI scores between the 2 groups (*P* > .05). On 14-day posttreatment, the intervention group exhibited significantly lower VAS and ODI scores and a higher JOA score compared to the control group (*P* < .05). On 28-day posttreatment, the intervention group continued to show significantly lower VAS and higher JOA scores than the control group (*P* < .05). Notably, both groups demonstrated significantly lower VAS and ODI scores and higher JOA scores on 14-day and 28-day compared to the baseline measurements (*P* < .05) (Table 2).

**Table 2:** Comparison of the VAS, JOA, and ODI scores between the 2 groups.

		Baseline	14 d	28 d
VAS	Intervention group	6.69 ± 1.09	2.86 ± 0.96*	2.59 ± 1.08#
	Control group	6.51 ± 1.82	4.06 ± 1.49*	3.06 ± 1.17#&
	*t*	−0.596	4.82	2.11
	*P* value	.553	<.001	.037
JOA	Intervention group	10.26 ± 3.61	19.98 ± 3.71*	20.04 ± 4.41#
	Control group	11.18 ± 3.20	18.00 ± 2.38*	18.29 ± 3.49#
	*t*	1.37	−3.21	−2.22
	*P* value	.175	.002	.029
ODI	Intervention group	33.80 ± 5.63	15.41 ± 3.96*	16.39 ± 5.04#
	Control group	32.47 ± 6.39	17.71 ± 5.00*	17.45 ± 5.71#
	*t*	−1.12	2.57	1.02
	*P* value	.267	.012	.309

* *P* < .05, Baseline vs 14 d.

#*P* < .05, Baseline vs 28 d.

&*P* < .05, 14 d vs 28 d.

### 3.3. Inflammation indicators of the 2 groups

Fourteen days following treatment, both groups exhibited a significant reduction (*P* < .001) in CRP, IL-6, and TNF-α compared to their baseline values. There were no significant differences in these baseline values between the groups (*P* > .05). On 14-day, the intervention group showed significantly lower levels of CRP and TNF-α than the control group (*P* < .01) (Table 3).

**Table 3:** Comparison of the CRP, IL-6 and TNF-α levels between the 2 groups.

		Baseline	14 d	*t*	*P* value
CRP (mg/L)	Intervention group	21.00 ± 13.08	8.50 ± 2.94	7.05	<.001
	Control group	26.10 ± 20.21	15.29 ± 7.42	3.96	<.001
	*t*	1.49	6.07		
	*P* value	0.141	<0.001		
IL-6 (pg/mL)	Intervention group	4.96 ± 3.59	2.66 ± 2.45	5.30	<.001
	Control group	4.76 ± 3.58	2.61 ± 2.33	5.21	<.001
	*t*	−0.273	−0.091		
	*P* value	0.785	0.928		
TNF-α (×10^3^ pg/mL)	Intervention group	2.46 ± 0.86	1.17 ± 0.28	11.30	<.001
	Control group	2.52 ± 1.05	1.40 ± 0.52	7.77	<.001
	*t*	0.27	2.77		
	*P* value	0.789	0.007		

### 3.4. Pressure pain threshold and pain tolerance threshold of the 2 groups

Fourteen days after treatment, both groups showed a significant increase in their pressure pain threshold and pain tolerance threshold values compared to baseline (*P* < .001). There were no significant differences in baseline values between the 2 groups (*P* > .05). On 14-day, pressure pain threshold of the intervention group was significantly higher than those of the control group (*P* = .002) (Table 4).

**Table 4:** Comparison of pressure pain threshold and pain tolerance threshold.

		Baseline	14 d	*t*	*P* value
Pressure pain threshold (kg/cm^2^)	Intervention group	3.76 ± 1.54	5.17 ± 1.91	−7.87	<.001
	Control group	3.26 ± 2.12	3.97 ± 1.96	−3.83	<.001
	*t*	−1.37	−3.13		
	*P* value	0.174	0.002		
Pain tolerance threshold (kg/cm^2^)	Intervention group	8.41 ± 2.33	9.54 ± 2.40	−4.99	<.001
	Control group	8.00 ± 2.64	8.80 ± 2.85	−3.49	.001
	*t*	−0.82	−1.42		
	*P* value	0.412	0.160		

## 4. Discussion

In TCM, LDH is categorized under lumbago, lumbar and leg pain, and arthralgia.^[31]^ This condition is often attributed to imbalances in the body’s qi, blood, meridians, and internal organs, with qi stagnation, blood stasis, and meridian obstruction being common etiologies. Although rehabilitation yields favorable outcomes for lumbar disc herniation patients, a substantial segment of the population increasingly chooses surgical intervention, especially as the patient demographic grows and skews younger, and as the severity of the condition escalates. However, surgical procedures inherently carry risks, and our research is primarily focused on the early alleviation of symptoms through conservative treatments. Patients experiencing pain and limitations in daily activities can potentially benefit from TCM. Accordingly, our controlled clinical trial investigated the effectiveness of Erxian decoction and auricular acupoint pressure bean therapy in managing symptoms in LDH patients.

Our research findings indicated that a combination of Erxian decoction and auricular acupoint pressure therapy was effective in treating LDH patients. This effectiveness was demonstrated by reduced pain levels, enhanced functional mobility, increased pain thresholds, and lowered serum inflammatory markers in patients. To our knowledge, this study represented the first instance of integrating Erxian decoction with auricular acupoint pressure for treating LDH patients. The primary active compounds of Erxian decoction, such as icariin, timosaponin BII, nystose, and curculigoside, exhibit diverse effects on osteoblasts and osteoclasts.^[32]^ Their impact includes enhancing osteoblast proliferation, reducing the number of multinucleated osteoclasts and tartrate-resistant acid phosphatase (TRAP) activity, and elevating alkaline phosphatase (ALP) activity.^[33]^ A study comparing the efficacy of Caltrate tablets and Erxian decoction reported that Erxian decoction significantly improved lumbar spine bone density.^[34]^ Additionally, a systematic review indicated that combining Erxian decoction with other treatments resulted in greater enhancement of lumbar spine bone density compared to the use of single medications like elcatonin, hormones, and salmon calcitonin.^[17,34–36]^ Bone loss and osteoporosis can contribute to lumbar spine pain and impede mobility.^[37]^ Consequently, Erxian decoction could mitigate osteoporosis risk and alleviate pain in LDH patients. However, our search yielded no studies addressing the connection between Erxian Tang and quality of life in LDH patients. We speculate that enhanced functional mobility correlates with reduced pain.

Auricular acupoint pressure with beans, an ancient Chinese medical technique dating back over 2000 years, has been reviewed for its efficacy. Systematic reviews indicated that targeting auricular acupressure points alleviated acute and chronic pain in various body areas, including pain following lumbar spine surgery.^[21,22]^ The research conducted by Yeh et al^[24]^ also included auricular acupressure therapy as a method for low back pain relief among the elderly population. It is crucial to recognize that auricular acupressure therapy involves selecting specific points based on the location of pain. Commonly, the Shenmen and subcortex acupoints are chosen for their correspondence to various areas of pain.^[21]^ Prior research has demonstrated that variations in pro-inflammatory cytokines, such as IL-6 and TNF-α, are linked to pain signaling pathways.^[38]^ It’s also been established that auricular acupressure can lower the expression of these inflammatory cytokines, thereby alleviating pain. Similar outcomes were observed in a previous controlled trial involving patients with low back pain and in our own experimental study.^[39]^ Santoro et al discovered that auricular acupressure primarily increased pain tolerance, rather than altering the minimum pain threshold.^[40]^ The experiment by Bui et al also revealed that applying pressure to the Shenmen auricular acupoint significantly raised the pain threshold in facial skin.^[41]^ These findings aligned with the outcomes observed in our own experiments.

Our study has several limitations: the sample size was relatively small, necessitating future expansion for more comprehensive results; our study population was limited to proactive patients, potentially indicating that all participants had relatively severe symptoms. This selection criterion introduced the possibility of bias in our findings; the data collection intervals were not extensive enough to thoroughly assess the long-term efficacy of the combination therapy.

## 5. Conclusion

In summary, the synergistic application of Erxian decoction and auricular acupoint pressure therapy in LDH patients significantly outperformed rehabilitation alone. This combination effectively alleviated pain, reduced inflammatory responses, and offered greater safety and clinical efficacy. Consequently, this integrative approach using 2 traditional Chinese medicinal therapies merits broader application and promotion.

## Author contributions

**Data curation:** Wei Feng, Xixi Du, Yuxin Zhao.

**Formal analysis:** Wei Feng, Xixi Du, Yuxin Zhao.

**Methodology:** Wei Feng, Xixi Du.

**Software:** Wei Feng, Xixi Du.

**Visualization:** Wei Feng, Xixi Du.

**Writing – original draft:** Wei Feng, Xixi Du, Yuxin Zhao.

**Conceptualization:** Yuxin Zhao.

**Funding acquisition:** Yuxin Zhao.
